# Broadband Surface Plasmon Lasing in One-dimensional Metallic Gratings on Semiconductor

**DOI:** 10.1038/s41598-017-08355-6

**Published:** 2017-08-11

**Authors:** Seung-Hyun Kim, Won Seok Han, Tae-Young Jeong, Hyang-Rok Lee, H. Jeong, D. Lee, Seung-Bo Shim, Dai-Sik Kim, Kwang Jun Ahn, Ki-Ju Yee

**Affiliations:** 1 Department of Physics, Chungnam National University, Daejeon, 34134 South Korea; 2 Electronics and Telecommunications Research Institute, Daejeon, 34129 South Korea; 3 Korea Research Institute of Standards and Science, Daejeon, 34113 South Korea; 4 Department of Physics and Astronomy, Seoul National University, Seoul, 08826 South Korea; 5 Department of Energy Systems Research and Department of Physics, Ajou University, Suwon, 16499 South Korea; 6 Korea Basic Science Institute, Daejeon, 34134 South Korea

## Abstract

We report surface plasmon (SP) lasing in metal/semiconductor nanostructures, where one-dimensional periodic silver slit gratings are placed on top of an InGaAsP layer. The SP nature of the lasing is confirmed from the emission wavelength governed by the grating period, polarization analysis, spatial coherence, and comparison with the linear transmission. The excellent performance of the device as an SP source is demonstrated by its tunable emission in the 400-nm-wide telecom wavelength band at room temperature. We show that the stimulated emission enhanced by the Purcell effect enables successful SP lasing at high energies above the gap energy of the gain. We also discuss the dependence of the lasing efficiency on temperature, grating dimension, and type of metal.

## Introduction

Surface plasmons (SPs) are collective electric charge density oscillations on metal surfaces^1, 2^. Because of their long propagation length along the metal dielectric interface travelling at nearly the speed of light and their ability to preserve coherence beyond the diffraction limit, optical information processing via SPs is very promising^3, 4^. Currently, SP sources with a high pulse repetition rate and broad spectral tunability are in great demand^5^. However, large ohmic losses due to field penetration through the metallic region need to be solved if the SP signal is to perform complex functions. One way of overcoming this issue is by incorporating additional gains to compensate the loss^6, 7^.

If the applied gain is larger than the loss in an SP cavity, it can lead to SPASER (SP amplification via stimulated emission of radiation) or SP lasing^8^. The phenomenon of SPASER is a well-established and straightforward method of generating coherent SP sources^9, 10^. Similar to conventional lasers^11^, SPASER operation can be achieved via the stimulated energy transfer from a gain medium to a subwavelength SP resonator (SPR) through feedback. The lasing SPASER proposed by Zheludev *et al*.^12^ is another type of SP source and, is accomplished by embedding a periodic array of SPRs on gain materials. In this approach, SP radiation is enabled by collectively oscillating mode currents generated in the SPRs. Recently, several groups have reported the production of lasing SPASERs, either through the use of individual nanoparticles coupled with gain media^9, 13–15^ or based on periodic structures^16–19^.

In this paper, we report SP lasing in a hybrid metal/semiconductor nanostructure consisting of periodic silver slit arrays on top of an InGaAsP layer. The lasing mode exhibits many characteristics of SPs, and can cover the broadband spectral range from 1180 to 1570 nm by changing the grating period. We investigate the dependence of SP lasing on the temperature, grating width, and grating material.

## Results and Discussion

**Figure 1:**
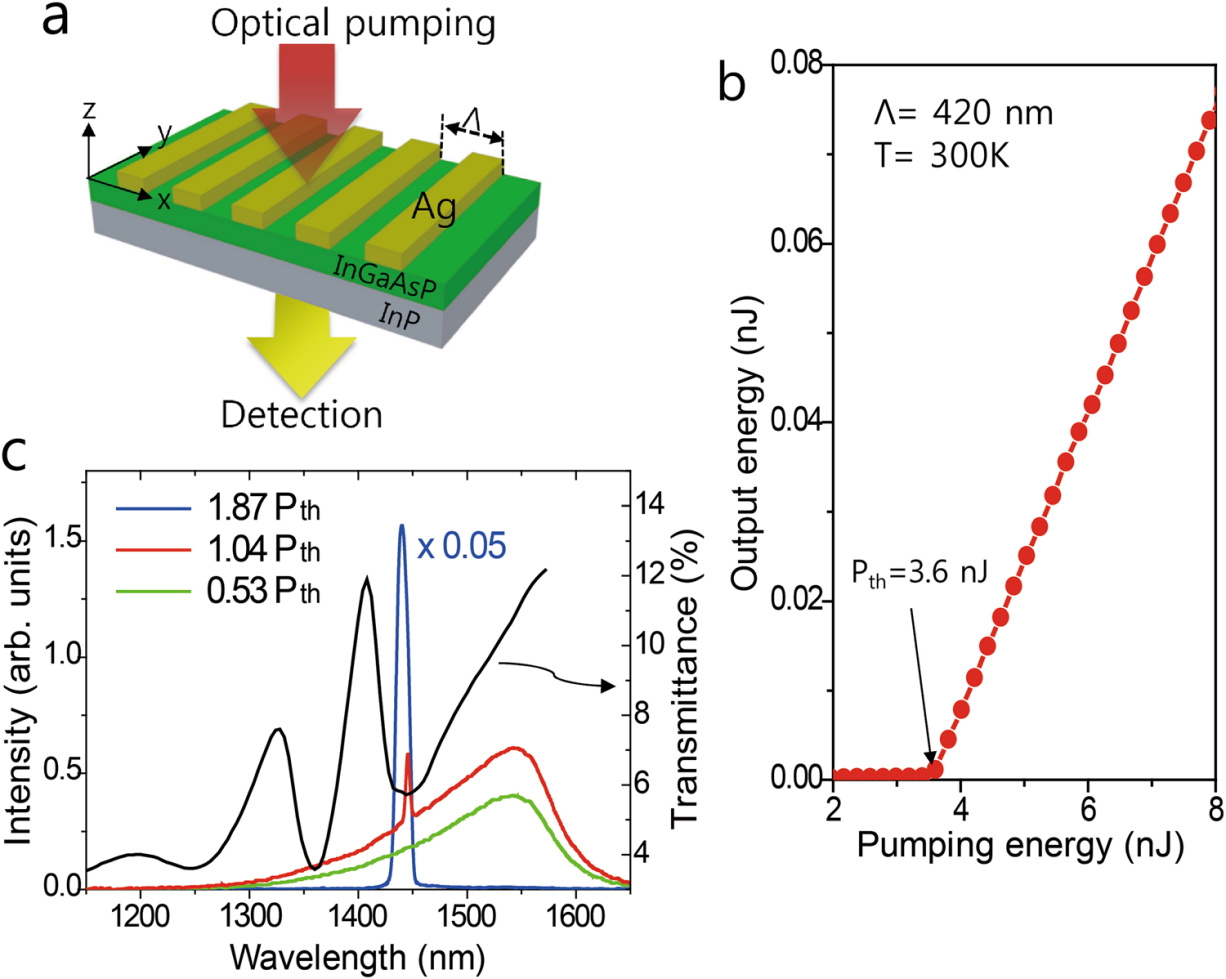
Demonstration of the SP lasing. (**a**) Schematic of the sample structure and experimental configuration. (**b**) Emission intensity as a function of the pump energy for a sample with Λ = 420 nm and *FF* = 0.6 at T = 300 K. (**c**) Emission spectrum measured for three different pump energies. For comparison, the linear transmission spectrum is plotted as a solid grey line. Lasing emission appears at the transmission dip caused by resonant SP excitation.

In order to demonstrate SP lasing, we have fabricated one-dimensional silver slit arrays on the top surface of a 700 nm thick layer of InGaAsP (Fig. 1a). The period Λ and metal fill factor *FF* of the slit array are adjusted such that it ensures efficient SP lasing. The electrons which are excited to the conduction band by a pump pulse energy *P* relax to the band edge through non-radiative processes. The emission energy as a function of pumping energy for the sample with Λ = 420 nm and *FF* = 0.6 at T = 300 K is presented in Fig. 1b. The drastic increase in the slope beyond a critical point indicates that the lasing is built-up above the threshold pump energy of *P*_*th*_ = 3.6 nJ and the slope efficiency is 1.7%. The spectral evolution of the emission is presented in Fig. 1c. While spectrally broad photoluminescence below the threshold (*P* = 0.53 *P*_*th*_) is caused by the spontaneous recombination of electron hole pairs in the InGaAsP region, a sharp peak by the stimulated emission emerges at a short-wavelength shoulder for *P* = 1.03 *P*_*th*_. This peak is predominant when the pump energy is sufficiently larger than the threshold energy (*P* = 1.87 *P*_*th*_). According to our previous studies with the same structure^20, 21^, the dips at around 1360 and 1440 nm in the linear transmittance (black line in Fig. 1c) are caused by the resonance of the waveguide and SP mode, respectively. By comparing the emission spectrum with the transmittance, we find that the narrow peak coincides with the SP resonance (*λ*_*sp*_ = 1.44 μm).

**Figure 2:**
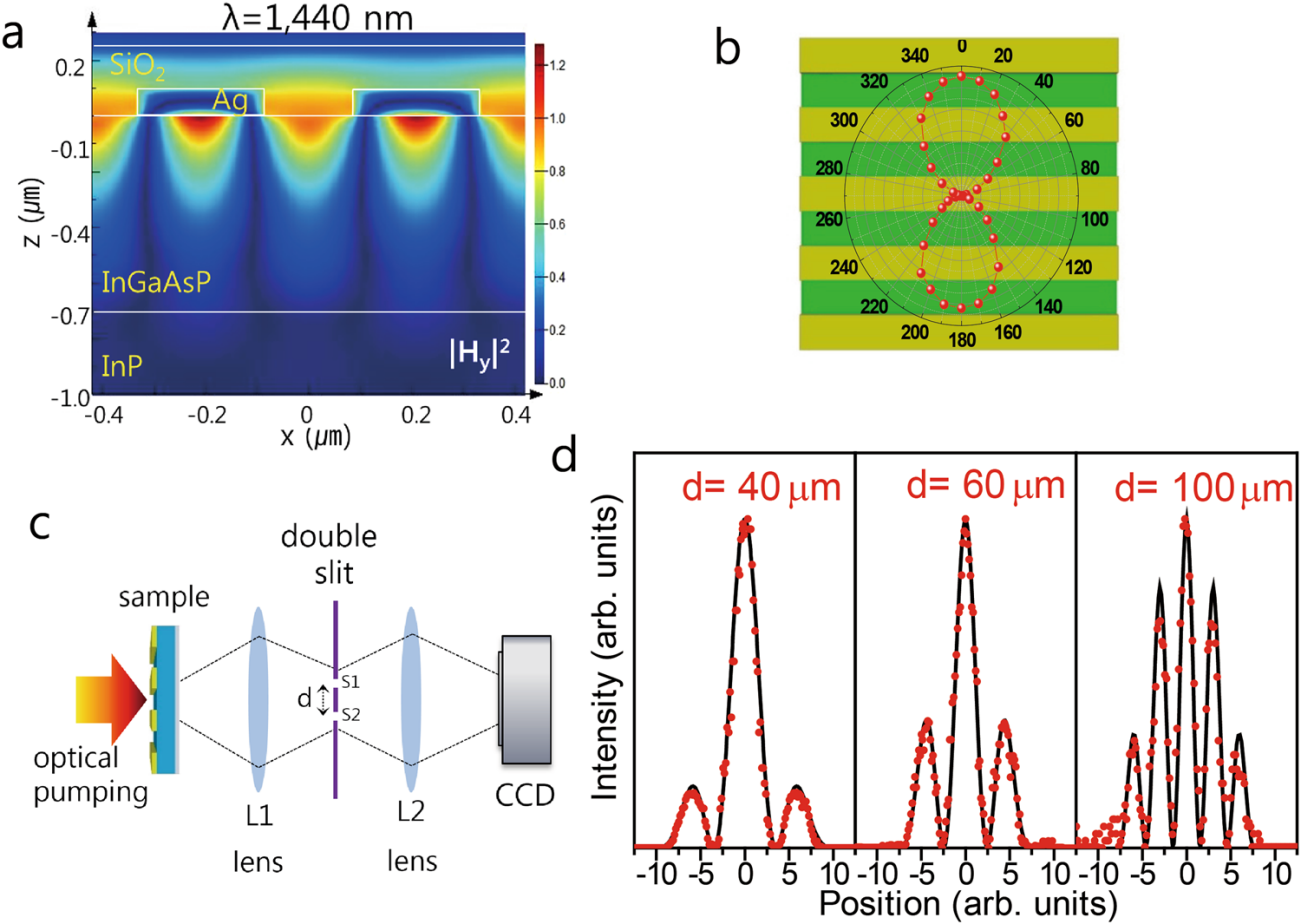
Characteristics of the SP lasing. (**a**) FDTD simulation of the electromagnetic fields at a grating structure with Λ = 420 nm induced by normally incident light at the lasing wavelength (1440 nm, TM polarization). (**b**) Polarization direction analysis of the SP lasing. (**c**) Schematic setup of Young’s double-slit experiment for spatial coherence measurements. (**d**) Diffraction patterns measured with a CCD camera for slit separations of 40, 60, and 100 μm. The solid lines are fitting curves given by Eq. (1).

The electromagnetic field scattered by the grating structure for the normal incidence was simulated using a commercial finite-difference time-domain (FDTD) program^22^, for which the dispersive relative permittivities of Ag^23^, InP^24^, and InGaAsP^25^ were used. The magnetic field profile at the lasing wavelength (1440 nm, transverse magnetic (TM) polarization) for the sample with Λ = 420 nm (Fig. 2a), indicates strong field confinement at the metal/semiconductor interface, while the field decays evanescently as the distance from the interface increases. By rotating the polarizer in front of the detector, we find that the lasing emission is polarized perpendicular to the slit length direction (Fig. 2b), corresponding to the TM mode. Both the polarization analysis and field profile prove that the lasing is based on SPs at the Ag/InGaAsP interface.

In order to study the spatial coherence of the lasing emission, we performed double-slit experiments, as shown in the schematic in Fig. 2c (see Methods). Here, the pump beam does not contribute to the signal because it is completely absorbed in the InP substrate. The diffracted light is expected to form an interference pattern on the CCD, which can be described by^26^

1 $$I(x)={I}_{1}(x)+{I}_{2}(x)+2{g}^{(1)}(r)\sqrt{{I}_{1}(x){I}_{2}(x)}\,\cos (\phi (x)).$$

*I*_1,2_(*x*) is the position-dependent intensity distribution on the CCD image plane, diffracted by slit 1 or 2, respectively, and *φ*(*x*) is the phase difference between them. The spatial coherence is evaluated from the first-order spatial correlation function *g* ^(1)^(*r*) for the spatial distance *r* at the grating. It is demonstrated from the vivid interference pattern in Fig. 2d up to the slit separation of 100 μm that the lasing emission contains a somewhat long spatial coherence.

The mechanism of SP lasing is basically similar to that of conventional distributed feedback lasers^27^. Additionally, the far-filed emission profile in Figure S1 of supplementary information exhibits a double-lobed pattern, which is a typical radiation property of distributed feedback lasers^28^. As predicted from the field profile in Fig. 2a, the SP mode exhibits resonance at a wavelength ${\lambda }_{{\rm{SP}}}={\rm{\Lambda }}\sqrt{{\varepsilon }_{Ag}{\varepsilon }_{d}/({\varepsilon }_{Ag}+{\varepsilon }_{d})}={\rm{\Lambda }}{n}_{e}$, where *ε*_*Ag*_ and *ε*_*d*_ are the dielectric constants of Ag and InGaAsP, respectively. The square root term was replaced by the effective refractive index *n*_*e*_. The SP waves generated via photoexcited carriers in InGaAsP are scattered by the periodic metal slits, where the waves interfere coherently if the Bragg condition of m*λ*_*B*_ = 2Λ*n*_*e*_ satisfied, where m is an integer number. While m = 1 corresponds to the edge emitting mode, the Bragg wavelength *λ*_*B*_ at the normally radiating lasing mode demonstrated in this study is determined by m = 2^29^.

**Figure 3:**
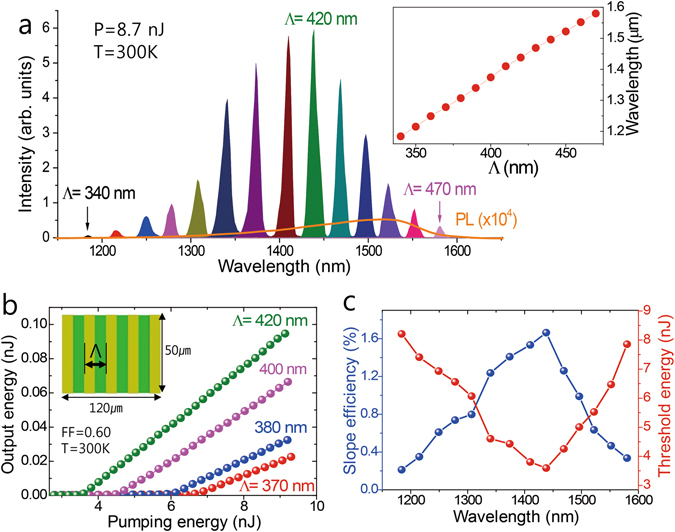
Emission wavelength tunability. (**a**) Lasing spectra for different slit periods Λ at a constant pump energy of 8.7 nJ. The peak positions are presented in the inset as a function of the period. (**b**) Output versus pumping energy curves obtained from gratings with different periods. (**c**) Lasing efficiencies (blue), defined as the linear slope of the output energy with respect to the pump energy, and threshold energies (red) for different slit periods, shown as a function of lasing wavelength.

Figure 3a illustrates the dependence of the lasing spectrum on the grating period. For a constant pump energy *P* = 8.7 nJ at T = 300 K, the peak wavelength is tuned from 1.18 to 1.57 μm by varying Λ from 340 to 470 nm (inset in Fig. 3a). Figure 3b indicates that the threshold energy and slope efficiency of the output versus pump energy strongly depend on the period. The sample with Λ = 420 nm exhibits the highest efficiency and lowest threshold energy. Assuming a pump photon absorbance of 78% in the InGaAsP layer, which was estimated from the FDTD simulation, the pump threshold of P_th_ = 3.6 nJ for Λ = 420 nm corresponds to the threshold carrier density of *N*_*th*_~5.7 × 10^18^ cm^−3^.

It is remarkable that the lasing succeeds over a range as broad as 400 nm or 30% Δλ/λ in the telecommunication band. In previous studies based on waveguide modes, distributed feedback lasers could reach a tuning range of 25 nm from the InGaAsP/InP system and 75 nm by using a fluorine polymer gain^30, 31^. Even for conventional diode lasers, multiple gains with different energy gaps were required to achieve a comparable broadness^32^. In the extreme case of Λ = 340 nm, SP lasing occurs at 1184 nm, which is energetically higher than the band edge of InGaAsP by more than one-third of the gap energy. In order to establish population inversion at such a high energy, the carrier density *N* must be high. However, the lasing condition will then become harsh because the accelerated carrier recombination is proportional to *N* ^2^ and the increased photonic loss is caused by free carrier absorptions. We estimate that the Purcell effect at the SP resonance contributes to the overcoming of those difficulties and, eventually enables SP lasing at high energies. If the gain enhancement by the Purcell effect accelerates the lasing build-up time, the efficiency will be less influenced by the slower spontaneous carrier recombination. In this regard, the lasing at high energies can be an evidence for the enhanced stimulated emission of SP waves. We note that the Purcell effect is stronger at the SP mode than at the waveguide mode, which is confirmed by the faster carrier recombination at the SP resonance (see Figure S2 in supplementary information) and can explain the survival of the SP mode in the lasing mode competition.

Figure 3b and c indicate that the threshold power is the lowest for the grating with Λ = 420 nm at a wavelength of 1440 nm, of which the energy is somewhat higher than the band edge (~1550 nm). This property is dissimilar to conventional semiconductor lasers where the lowest threshold is close to the band edge. This property of SP lasing comes from the large propagation loss of the SP wave. In order to reach lasing despite the SP loss, a large gain is required and this can be easier at moderately high energies with a considerable density of states.

**Figure 4:**
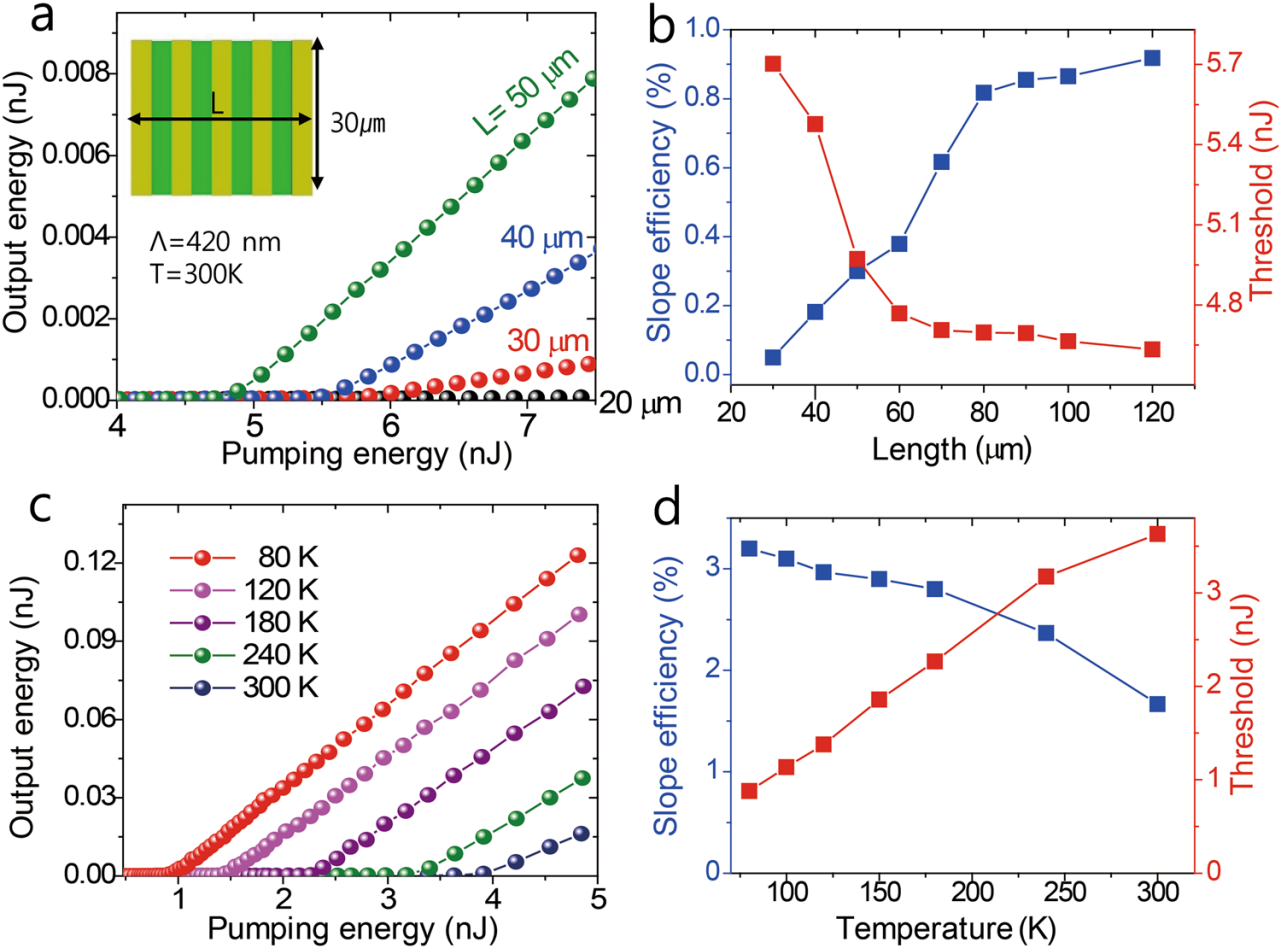
Temperature and grating length dependence. (**a**) Output versus pumping energy curves obtained from grating structures with a fixed period of Λ = 420 nm and a length varying from 20 to 50 μm. (**b**) Lasing efficiencies (blue) and threshold energies (red) for different grating lengths. (**c**) Temperature-dependent performance of a device with Λ = 420 nm. (**d**) Lasing efficiency (blue) and threshold energy (red) as a function of sample temperature.

As the SP wave propagates along the metal grating, it is partially reflected and out-coupled from the slits. If the effective reflectivity of *R*_eff_ is assumed and *L* represents the cavity length, the threshold condition can be described as ${R}_{{\rm{eff}}}\times {e}^{(g-\gamma )L}=1$, where *g* and *γ* are the gain and loss coefficient, respectively. This simplified model predicts a smaller threshold gain for a longer cavity. We tested the lasing performance by varying the grating length. It is consistent with the prediction that the threshold energy and efficiency in Fig. 4a and b improve with the grating length. The efficiency saturation for lengths larger than 70 μm can be explained by the finite spot size of the pump beam.

With the electron distribution following Fermi–Dirac statistics, a specific threshold gain is easily achieved at low temperatures, and the metallic loss of the SP propagation is also smaller as the temperature is lower. Figure 4c and d show the temperature dependent threshold energy and lasing efficiency for the sample with Λ = 420 nm. They demonstrate that the threshold energy decreases by a factor of four and the efficiency improves by a factor of two as the sample is cooled from 300 to 80 K.

**Figure 5:**
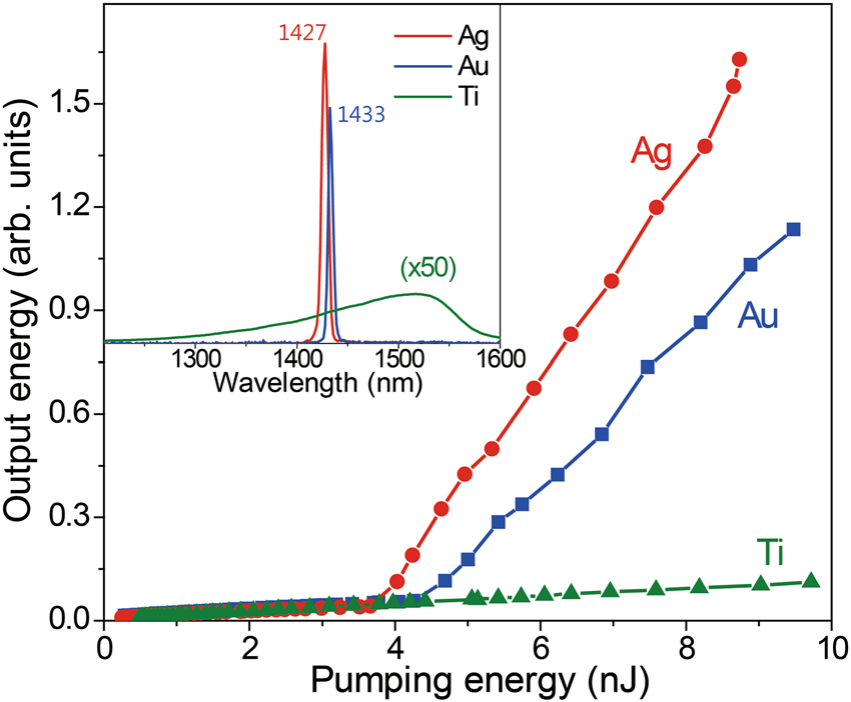
SP lasing from slits of different metals. Output versus pumping energy curves for metal gratings with Λ = 420 nm, made from different materials of Ag, Au, and Ti. The inset shows the emission spectra of the different metal slits at a pumping energy of 6 nJ.

In order to further clarify that the lasing originates from SPs, we measure the samples fabricated with different metals. Figure 5 shows the emission versus pump characteristic curves for the metal gratings made of Ti, Au, and Ag. In contrast to the case of Au or Ag slits, the Ti slit array does not exhibit lasing performance. The superiority in efficiency of the Ag slit array over the Au slit array is in a good agreement with the general tendency that Ag exhibits a smaller loss at the considered wavelength. The blue-shifted lasing wavelength of the Ag slits with respect to that of the Au slits results from the different dielectric constants of the metals, which also results in the shift of the transmission dip in the FDTD calculation (see Figure S3 in supplementary information). The faster radiative decay rate of photoexcited carriers in the sample with the Ag slit, in comparison to those with the Au or Ti slit, further supports that the superior SP lasing by the Ag slit is attributed in part to the stronger Purcell enhancement as demonstrated in Figure S1 of supplementary information.

## Conclusion

We demonstrate that one-dimensional slit arrays of Ag coupled with an InGaAsP layer enables SP lasing over a broadband spectral range. The period dependent systematic tuning over a 400-nm-wide wavelength range in the telecommunication band is achieved by the efficient gain transfer from the carriers in the InGaAsP and the SP mediated Purcell enhancement of the stimulated emission. Because of the routine fabrication process and wide tunability, the SP source in this study can be incorporated into diverse photonic applications. We expect that further studies on different semiconductor materials and improved cavity designs will enable this type of SP source to operate at visible wavelengths and to exhibit higher efficiencies and compactness.

## Methods

### Sample fabrication

A 700-nm-thick epilayer of InGaAsP with a band edge near 1.55 μm at room temperature was grown on an InP substrate by metal–organic chemical vapor deposition. Then, one-dimensional slit gratings of 100-nm-thick Ag were fabricated on top of the InGaAsP layer using e-beam lithography. Finally, a 250 nm thick SiO_2_ layer overcoat was added to prevent oxidation of the silver layer. The grating period was varied from 340 to 500 nm with a metal coverage of 60%. The overall dimension of each grating was typically 50 × 120 μm^2^, with the longer axis perpendicular to the slit direction.

### Optical characterization

The metal/semiconductor nanostructures were optically pumped by femtosecond (fs) pulses from a Ti:sapphire oscillator, which have a spot diameter of about 60 μm, centre wavelength of 800 nm, and pulse duration of 300 fs. The emission spectrum and power were measured using an InGaAs spectrometer and Ge photodiode, respectively. The temperature-dependent experiments were performed with the sample in a cryostat.

### Double-slit diffraction

For the double-slit diffraction measurements, the emission from the sample was focused onto a double-slit located between two lenses L1 and L2 (Fig. 2c). The separation d between the two 30-μm aperture slits (s1, s2) were varied from 40 μm to 100 μm, and the far-field pattern resulting from the diffraction at the double-slit was measured using a CCD camera. The beam spot size at the plane of the double-slit and that at the grating was comparable, implying that the slit separation d could be projected to a distance *r* on the grating.

## Electronic supplementary material

Supplementary information
