# Supplementary material for: Broadband Surface Plasmon Lasing in One-dimensional Metallic Gratings on Semiconductor
Source: Sci Rep. 2017 Aug 11;7:7907. doi: 10.1038/s41598-017-08355-6 (PMC5554227; doi:10.1038/s41598-017-08355-6)
Supplement: Supplementary file 1 — Supplementary information [file 41598_2017_8355_MOESM1_ESM.doc]

*Supplementary Information*

Broadband Surface Plasmon Lasing in One-dimensional Metallic Gratings on Semiconductor

Seung-Hyun Kim^1^, Won Seok Han^2^, Tae-Young Jeong^1^, Hyang-Rok Lee^1^, H. Jeong^1^, D. Lee^1^, Seung-Bo Shim^3^, Dai-Sik Kim^4^, Kwang Jun Ahn^5^, and Ki-Ju Yee^1^

^1^Department of Physics, Chungnam National University, Daejeon 305-764, Korea

^2^Electronics and Telecommunications Research Institute, Daejeon 305-700, Korea

^3^Korea Research Institute of Standards and Science, Daejeon 305-340, Korea

^4^Department of Physics and Astronomy, Seoul National University, Seoul 151-747, Korea

^5^Department of Energy Systems Research and Department of Physics, Ajou University, Suwon 443-749, Korea


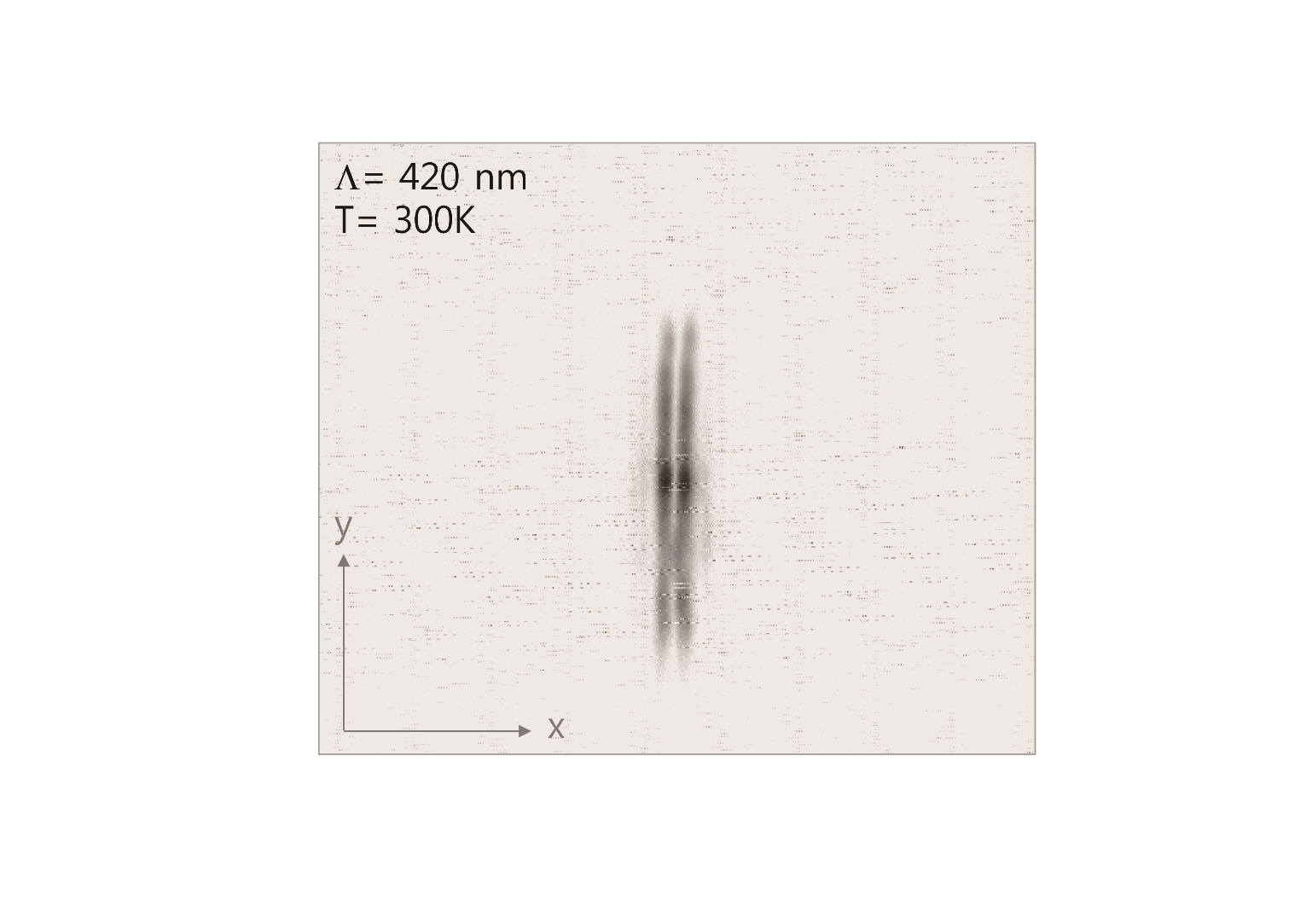


Figure S1. Far-field emission pattern of the SP lasing obtained from the Ag slits with Λ=420 nm.


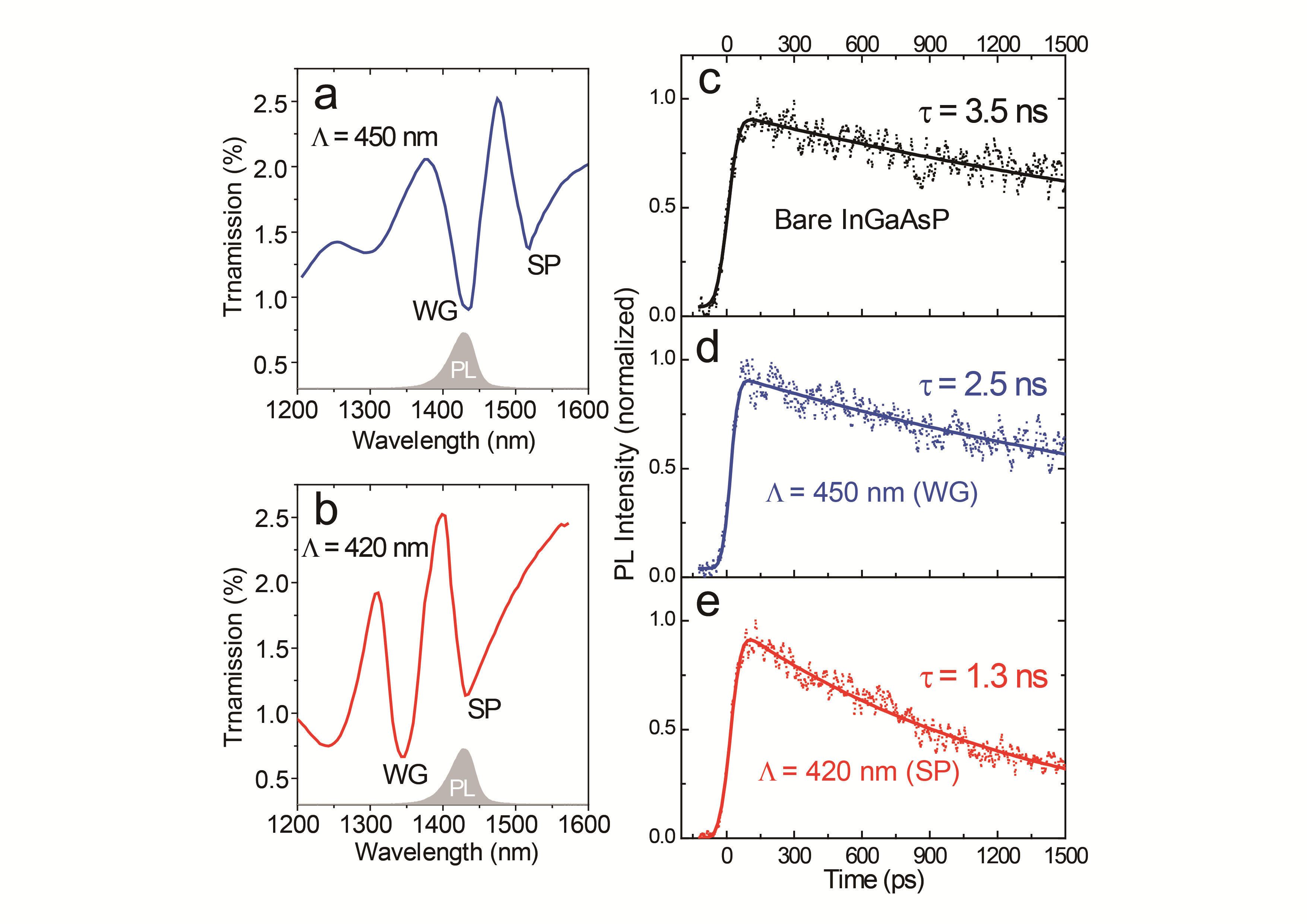


**Figure S2.** Experimentally measured transmissions of two Ag slit array samples of (a) Λ =450 nm and (b) Λ =420 nm where the WG and the SP mode are respectively matched to the PL peak position. The time-resolved photoluminescence signal of (c) a bare InGaAsP and that of the sample whose emission energy is spectrally aligned (d) to the WG (Λ =450nm) and (e) to the SP mode (Λ=420nm). Time-resolve photoluminescence was measured using a streak camera (C1587, Hamamatsu) with 10 ps temporal resolution and a Ti:sapphire laser as an optical pump source.


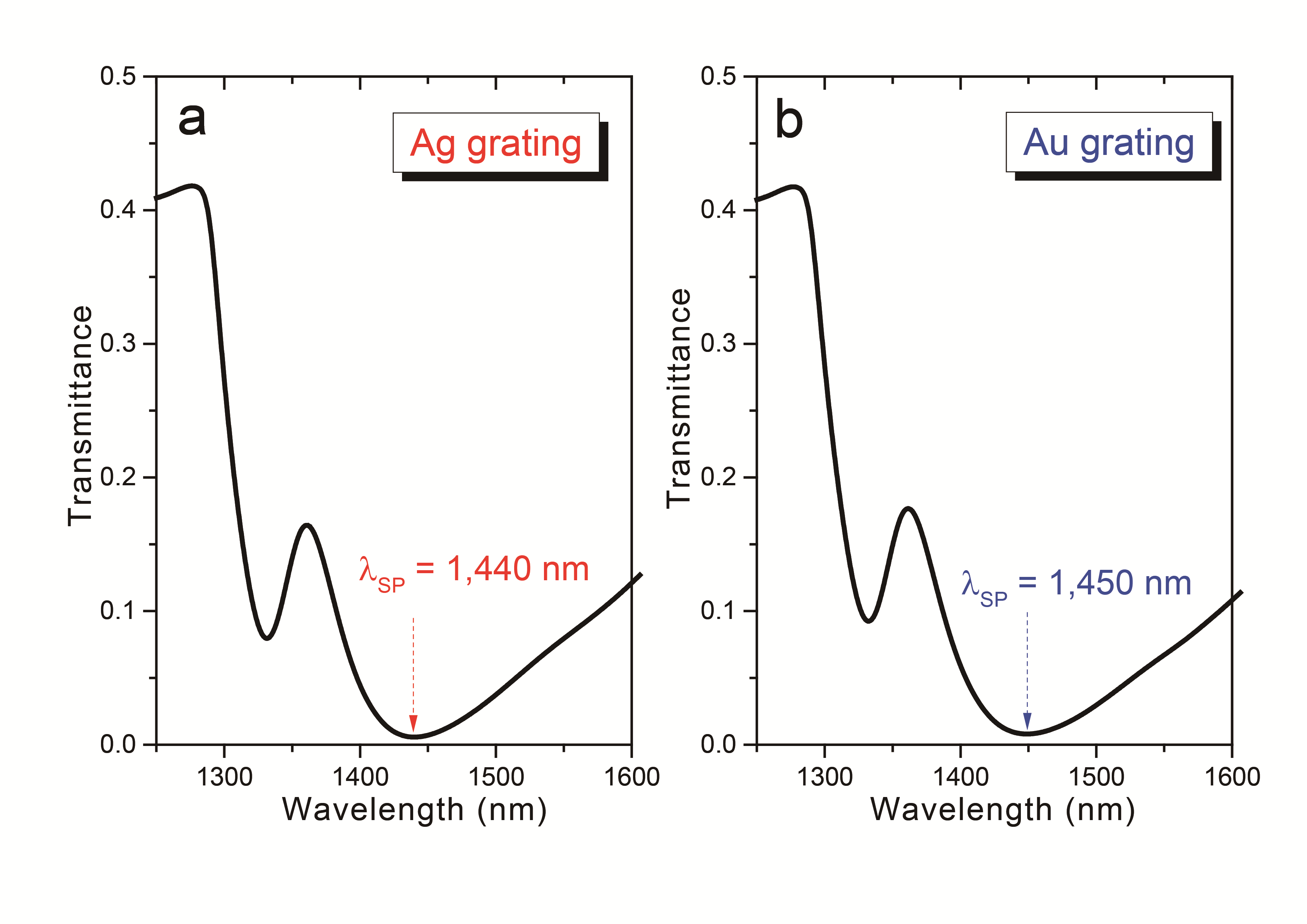


**Figure S3.** FDTD simulation of linear transmittance spectra through (a) the Ag and (b) Au grating with Λ=420 nm. The arrows indicate the transmittance dips from the SP resonance.


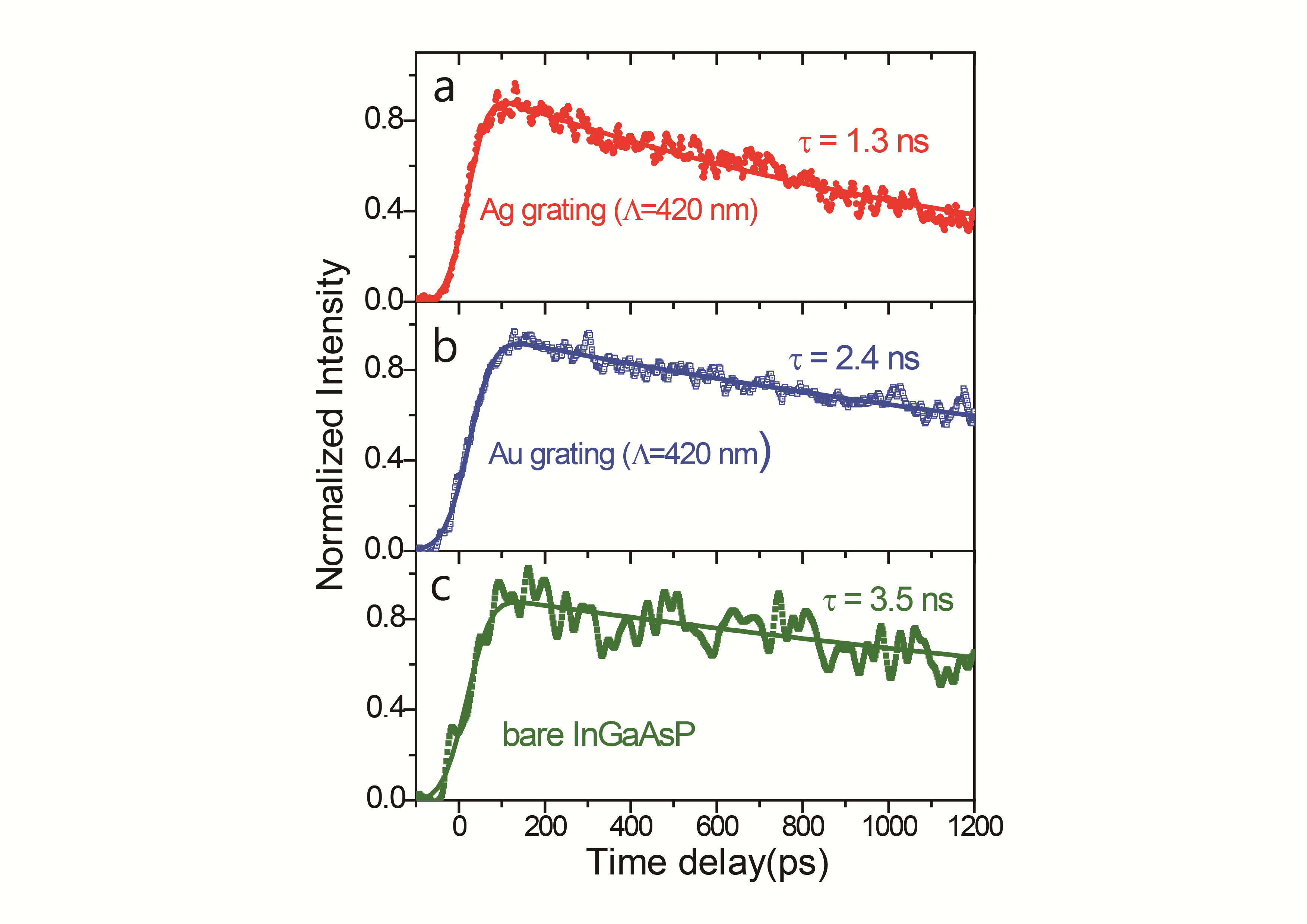


**Figure S4.** Time-resolved photoluminescence from (a) the sample with Ag grating, (b) that with Au grating, and (c) a bare InGaAsP at a temperature of 80 K. The pump energy of 0.28 nJ used in this experiment is below the lasing threshold.
